# Predictors and Characteristics of Hair Loss Among Users of GLP‐1 Receptor Agonists: A Cross‐Sectional Analysis

**DOI:** 10.1111/jocd.70835

**Published:** 2026-03-31

**Authors:** Yahya Argobi, Norah Saad Jadaan, Hind Bader Alshalhoob, Manar Saleh Alyousef, Ghaid Mohammed Alotaibi, Hussain Sami Alwesaibie, Ibtihal Sultan M. Alshehri

**Affiliations:** ^1^ King Khalid University Abha Saudi Arabia; ^2^ Majmaah University Al Majmaah Saudi Arabia; ^3^ King Fahad Specialist Hospital Saudi Arabia; ^4^ King Saud Bin Abdulaziz University for Health Sciences Saudi Arabia; ^5^ Almoosa Specialist Hospital Saudi Arabia

**Keywords:** clinical management, gender differences, GLP‐1 receptor agonists, hair loss, Mounjaro, semaglutide, side effects, telogen effluvium, tirzepatide, weight loss

## Abstract

**Background:**

The increasing use of GLP‐1 receptor agonists for weight loss and metabolic improvements has led to the recognition of various side effects. One potential, yet underexplored, adverse effect is hair loss. This study aims to investigate the frequency, characteristics, and predictors of hair loss in patients using GLP‐1RAs.

**Methods:**

This cross‐sectional study, conducted uniquely in Saudi Arabia from January to June 2025, involved 254 participants who had used GLP‐1RAs (Mounjaro, Ozempic, Saxenda, or Victoza) for weight loss purposes. Data were collected through structured questionnaires, ensuring comprehensive coverage of demographic information, clinical characteristics, and details of hair loss, including timing, severity, and progression. The data analysis was conducted using SPSS v29.

**Results:**

A total of 254 participants were included in the study, with the majority being female (71.3%) and an average age of 33.28 ± 9.73 years. Hair loss characteristics by injection type revealed no significant differences in prevalence (*p* = 0.116). However, severe hair loss was significantly more common in Mounjaro (43.4%) and Saxenda (42.9%) users compared to other groups (*p* < 0.001). Multivariable logistic regression analysis identified gender, marital status, and injection type as significant predictors of hair loss. Males were less likely to report hair loss compared to females (adjusted OR = 0.36, *p* = 0.003). Married participants had the highest odds of experiencing hair loss (adjusted odds ratio = 9.94, *p* = 0.004), and Mounjaro users had significantly higher odds of hair loss compared to the users of other injection types (adjusted odds ratio = 3.02, *p* = 0.009). The duration of injection use did not significantly predict hair loss, although a trend toward higher odds was noted for those using injections for 6–12 months (adjusted odds ratio = 3.50, *p* = 0.053).

**Conclusion:**

Our study highlights the significant side effects of hair loss associated with GLP‐1RA therapy, particularly in female patients and those using Mounjaro. Although this type of hair loss is typically non‐scarring and reversible, it can lead to psychological distress and affect treatment adherence. These conclusions highlight the need for clinicians to be aware of this potential side effect and to provide appropriate support and guidance to their patients.

## 1 Introduction

The use of weight loss medications has seen a marked increase in recent years as individuals strive not only for improved body composition but also for better overall health outcomes. Among these medications, glucagon‐like peptide‐1 receptor agonists (GLP‐1RAs) have gained particular prominence due to their effectiveness in achieving significant weight reduction and improving metabolic parameters in patients with type 2 diabetes mellitus and obesity [1, 2]. GLP‐1RAs including Mounjaro, Ozempic, Saxenda, or Victoza are increasingly used in clinical practice and have transformed the therapeutic landscape for metabolic diseases [3, 4].

However, as the prescription of GLP‐1RAs grows, attention is also turning to their side effect profiles. Gastrointestinal symptoms such as nausea and vomiting remain the most commonly reported adverse effects [5]. Injection site reactions and, more rarely, cutaneous manifestations—including hypersensitivity reactions and autoimmune blistering disorders—have also been documented [6]. Despite this growing body of knowledge, one area that remains understudied is the occurrence of hair loss associated with GLP‐1 receptor agonist (GLP‐1RA) therapy.

Hair loss whether due to telogen effluvium, androgenetic alopecia, or other mechanisms is a significant concern for many patients, impacting the quality of life and potentially influencing adherence to medication regimens. Although some case reports and small studies have noted the potential for drug‐induced alopecia in association with antidiabetic therapies, including teneligliptin and GLP‐1RAs, the true prevalence and risk factors for this phenomenon remain unclear [7]. Of particular interest, recent pooled analyses from large clinical trials have reported an increased frequency of drug‐induced alopecia with characteristics similar to telogen effluvium, among users of GLP1 for weight management, with most cases being mild, transient, and correlating with rapid weight reduction rather than the direct pharmacological effect of the drug itself [8].

Despite increasing anecdotal and clinical evidence, the literature still lacks comprehensive, real‐world data evaluating the predictors, types, and timing of hair loss in patients using GLP‐1RAs—especially outside of tightly controlled clinical trial environments [9, 10]. With the recent approval and increasing utilization of new agents, such as Mounjaro, which have demonstrated impressive weight loss outcomes, it is essential to systematically assess the potential cutaneous and dermatologic side effects including alopecia of these therapies [4].

The present study aims to investigate the frequency, characteristics, and predictors of hair loss among users of GLP‐1 receptor agonists, with a particular focus on Mounjaro. By examining demographic and clinical risk factors, types of hair loss, and the temporal relationship to therapy, this study aims to fill an essential gap in the literature and offer guidance for clinicians and patients on the dermatological safety of these increasingly used medications.

## 2 Material and Methods

This was a cross‐sectional study conducted in Saudi Arabia, from January to June 2025, involving adults who had used GLP‐1RAs for weight reduction. Data were collected from 254 participants attending clinics for weight loss. Eligible participants were aged 18 years or older, had used GLP‐1RA injections (Mounjaro, Ozempic, Saxenda, or Victoza) for at least one month, and provided informed consent. Individuals with pre‐existing scarring alopecia or on concurrent medications known to cause hair loss (such as chemotherapy) were excluded.

Data for this study were collected using a structured questionnaire administered to participants, which captured a broad range of variables pertinent to the investigation of hair loss among users of GLP‐1RA. Demographic information, including age, sex, nationality, marital status, and employment status, was collected, along with anthropometric data obtained from self‐reported height and weight. Participants were also asked to report their clinical characteristics, including current health conditions (such as hormonal disorders, polycystic ovary syndrome, anemia, thyroid disease, any history of telogen effluvium, and ferritin status). Detailed information regarding GLP‐1RA use was collected, encompassing the specific type of GLP‐1RA, the primary indication for its prescription (such as weight loss, diabetes management, or PCOS), and the duration of use (categorized as less than 3 months, 3–6 months, 6–12 months, or greater than 12 months). The questionnaire further explored participants' hair care practices, including their hair type, washing frequency, and the use of various hair products, such as dyes, straighteners, extensions, and curl enhancers.

Assessment of hair loss was based on self‐reported responses to items inquiring about current hair loss status (yes/no), timing of onset about the initiation of GLP‐1RA injections (chronic, within the first month, 1–3 months, 3–6 months, more than 6 months, or no change), severity of hair loss (mild, moderate, or severe), and progression over time (improved, stabilized, worsened, or unchanged). Additional questions addressed the type of hair fall (whether from the roots or due to splitting) and the approximate number of hairs lost daily (< 50, 50–100, 100–150, 150–200, and > 200).

For statistical analysis, SPSS v29 was used, and descriptive statistics (means, standard deviations, frequencies, and percentages) were used to summarize participant characteristics and features of hair loss. Comparative analyses were performed using the chi‐square test or Fisher's exact test for categorical variables (such as the association between injection type and hair loss prevalence or severity). In contrast, one‐way ANOVA or independent *t*‐tests were used for continuous variables. To identify independent predictors of hair loss among GLP‐1RA users, a multivariable logistic regression analysis was conducted, with hair loss (yes/no) as the dependent variable. Independent variables included gender, marital status, type of injection, and duration of GLP‐1RA use. Adjusted odds ratios (aOR) and 95% confidence intervals (CIs) were calculated, with statistical significance defined as *p* < 0.05.

## 3 Results

Table 1 presents the demographic and baseline characteristics of the participants. A total of 254 participants were included, with the majority of the sample being female (181, 71.3%), while males comprised 73 (28.7%). The mean age of the participants was 33.28 ± 9.73 years. The average height was 161.47 ± 18.0 cm, and the mean body weight was 77.86 ± 16.17 kg. Most participants were Saudi nationals (243, 95.7%), with a small proportion being non‐Saudi (11, 4.3%). Regarding marital status, 153 (60.2%) were married, 92 (36.2%) were single, and 9 (3.5%) were divorced. In terms of employment, 162 (63.8%) were employed.

**TABLE 1:** Demographic, clinical, and hair care characteristics of study participants (*n* = 254).

Variable	Value	%
Gender		
Male	73	28.7%
Female	181	71.3%
Age (mean ± SD)	33.28 ± 9.73	
Height (cm)	161.47 ± 18.0	
Weight (Kg)	77.86 ± 16.17	
Nationality		
Saudi	243	95.7%
Non‐Saudi	11	4.3%
Marital Status		
Single	92	36.2%
Married	153	60.2%
Divorced	9	3.5%
Job Status		
Not working	92	36.2%
Employed	162	63.8%
Type of Injection Used		
Victoza	1	0.4%
Saxenda	15	5.9%
Ozempic	50	19.7%
Mounjaro	188	74%
Duration of Injection Use		
< 3 months	78	30.7%
3–6 months	104	40.9%
6–12 months	48	18.9%
> 12 months	24	9.4%
Indication of Injection		
Diabetes	13	12.2%
Weight loss	121	87.0%
PCOS	2	0.8%
Current condition		
No condition	87	34.3%
Hormonal issues	42	16.5%
PCOS	34	13.4%
Moderate/severe anemia	33	13.0%
Thyroid disease	20	7.9%
Low ferritin	4	1.6%
Telogen effluvium (< 1 year)	34	13.4%
Hair Type		
Curly	52	20.5%
Dry	44	17.3%
Straight	73	28.7%
Wavy	85	33.5%
Hair Washing Frequency		
Daily	62	24.4%
Twice weekly	156	61.4%
Once weekly	32	12.6%
Rarely	4	1.6%
Use of Hair Products		
None	141	55.5%
Curl enhancers	8	3.1%
Hair dye	75	29.5%
Extensions	4	1.6%
Hair straighteners	14	5.5%
Straighteners + dye	12	4.7%

Among the weight‐reduction injections used, Mounjaro was the most common (74.0%), followed by Ozempic (19.7%), Saxenda (5.9%), and Victoza (0.4%). The duration of injection use varied, with 104 (40.9%) using injections for 3 to 6 months, 78 (30.7%) for less than 3 months, 48 (18.9%) for 6 to 12 months, and 24 (9.4%) for more than 12 months. The primary indication for injection use was weight loss 121 (87.0%), followed by diabetes 13 (12.2%) and polycystic ovary syndrome (PCOS) 2 (0.8%).

Regarding current health conditions, 87 (34.3%) reported having no underlying condition. Others reported hormonal issues 42 (16.5%), PCOS 34 (13.4%), moderate to severe anemia 33 (13.0%), thyroid disease 20 (7.9%), telogen effluvium within the past year 34 (13.4%), and low ferritin levels 4 (1.6%). Hair types reported included wavy 85 (33.5%), straight 73 (28.7%), curly 52 (20.5%), and dry 44 (17.3%). Most participants reported washing their hair twice a week 156 (61.4%), followed by daily washing 62 (24.4%), once weekly 32 (12.6%), and rarely 4 (1.6%). Over half of the participants, 141 (55.5%), reported no use of hair products, while others used hair dye (75, 29.5%) and hair straighteners (14, 5.5%).

Among the participants, the majority reported experiencing current hair loss (193, 76.0%), while 61 (24.0%) did not report hair loss. Of the 193 patients, 75 (38.8%) noticed it within 1 to 3 months after starting injections, 43 (22.3%) had chronic hair loss predating the injection use, 29 (15%) experienced hair loss within the first month, 25 (13%) within 3 to 6 months, and 4 (2.1%) after more than 6 months. A small proportion, 17 (8.8%), reported no change in hair status.

In terms of severity, 84 (43.5%) described their hair loss as moderate, 69 (35.8%) as severe, and 40 (20.7%) as mild. The progression of hair loss over time varied, with 92 (48.2%) reporting worsening hair loss, 77 (40.3%) noting stabilization, 20 (10.5%) reporting improvement, and 2 (1.0%) indicating no specific duration. Most participants (166, 86%) reported hair falling out from the roots, while 27 (14%) reported hair‐splitting. Daily hair fall volume was reported as follows: 57 (29.5%) lost 50–100 hairs, 46 (23.9%) lost 100–150 hairs, 37 (19.2%) lost fewer than 50 hairs, 31 (16%) lost 150–200 hairs, and 22 (11.4%) lost more than 200 hairs per day. Table 2.

**TABLE 2:** Hair loss characteristics among participants reporting hair loss.

Variable	Category	*n*	%
Current Hair Loss	No	61	24.0%
	Yes	193	76.0%
Hair Loss Onset	Chronic before injection	43	22.3%
	Within the first month	29	15.0%
	After 1–3 months	75	38.8%
	After 3–6 months	25	13.0%
	After 6+ months	4	2.1%
	Onset timing not specified	17	8.8%
Severity	Mild	40	20.7%
	Moderate	84	43.5%
	Severe	69	35.8%
Progression Over Time	Got better	20	10.5%
	Stabilized	77	40.3%
	Got worse	92	48.2%
	Not specified	2	1.0%
Hair Fall Type	From roots	166	86.0%
	Splitting	27	14.0%
Approx. Hairs Lost Daily	< 50	37	19.2%
	50–100	57	29.5%
	100–150	46	23.9%
	150–200	31	16.0%
	> 200	22	11.4%

Table 3 presents a comparative analysis of hair loss characteristics across different types of weight‐reduction injections. Among Mounjaro users, 136 (72.3%) reported hair loss, compared to 42 (84.0%) of Ozempic users, 14 (93.3%) of Saxenda users, and 1 (100%) of the single Victoza user. Although differences in hair loss prevalence were observed, the association was not statistically significant (*p* = 0.116). The onset of hair loss also varied by the type of injection. The most common onset period among Mounjaro users was within 1–3 months (54, 39.7%), similar to that of Ozempic users (17, 40.5%). Chronic hair loss before injection was most frequent among Saxenda users (5, 35.7%). Hair loss onset beyond 6 months was rare across all groups. No statistically significant differences in onset timing were found (*p* = 0.642).

**TABLE 3:** Comparison of hair loss characteristics by type of injection used among participants.

Variable	Victoza	Saxenda	Ozempic	Mounjaro	*P*
Hair Loss Status					
No	0 (0.0%)	1 (6.7%)	8 (16%)	52 (27.7%)	0.116
Yes	1 (100%)	14 (93.3%)	42 (84%)	136 (72.3%)	
Hair loss onset					
Chronic before injection	0	5 (35.7%)	12 (28.6%)	26 (19.1%)	0.642
Within the first month	0	3 (21.4%)	5 (11.9%)	21 (15.4%)	
After 1–3 months	0	4 (28.6%)	17 (40.5%)	54 (39.7%)	
After 3–6 months	1 (100%)	1 (7.1%)	3 (7.1%)	20 (14.7%)	
After 6+ months	0	0	1 (2.4%)	3 (2.2%)	
No changes	0	1 (7.1%)	4 (9.5%)	12 (8.8%)	
Severity					
Mild	1 (100%)	1 (7.1%)	13 (31.0%)	25 (18.4%)	
Moderate	0	7 (50%)	25 (59.5%)	52 (38.2%)	< 0.001
Severe	0	6 (42.9%)	4 (9.5%)	59 (43.4%)	
Progression over time					
Got better	0	1 (7.1%)	5 (11.9%)	15 (11.0%)	
Stabilized	0	7 (50%)	19 (45.2%)	51 (37.5%)	0.953
Got worse	1 (100%)	6 (42.9%)	17 (40.5%)	68 (50%)	
No change	0	0	1 (2.4%)	2 (1.5%)	
Hair fall type					
From roots	1 (100%)	13 (92.9%)	36 (85.7%)	116 (85.3%)	0.857
Splitting	0	1 (7.1%)	6 (14.3%)	20 (14.7%)	
Approx. Hairs Lost Daily					
< 50	1 (100%)	1 (7.1%)	10 (23.8%)	25 (18.4%)	
50–100	0	5 (35.7%)	15 (35.7%)	37 (27.2%)	
101–150	0	3 (21.4%)	10 (23.8%)	33 (24.3%)	0.259
151–200	0	1 (7.1%)	3 (7.1%)	27 (19.9%)	
> 200	0	4 (28.6)	4 (9.5%)	14 (10.3%)	

Hair loss severity differed significantly among groups (*p* < 0.001). Severe hair loss was most prevalent in Mounjaro users (59, 43.4%) and Saxenda users (6, 42.9%), while Ozempic users most frequently reported moderate severity (25, 59.5%). The only Victoza user reported mild hair loss.

Progression of hair loss over time showed no significant differences across injection types (*p* = 0.953). In all groups, the majority reported that hair loss had worsened over time, particularly among Mounjaro users (68, 50.0%) and Ozempic users (17, 40.5%). Regarding the type of hair fall, most participants across all injection types reported shedding from the roots, with slight variation (e.g., Mounjaro: 85.3%, Saxenda: 92.9%, *p* = 0.857). Daily hair fall volume showed no statistically significant difference among the groups (*p* = 0.259). Hair shedding of 50–100 hairs per day was the most frequently reported range, particularly among users of Saxenda (35.7%) and Ozempic (35.7%). A higher volume of hair loss (> 200 hairs/day) was reported by 28.6% of Saxenda users, compared to 9.5% of Ozempic and 10.3% of Mounjaro users. A single Victoza user reported hair loss of fewer than 50 hairs per day.

Table 4 presents the results of a multivariable logistic regression analysis identifying predictors of hair loss among injection users. Gender was significantly associated with hair loss, with males having lower odds of reporting hair loss compared to females (adjusted odds ratio = 0.36, 95% confidence interval [0.18, 0.70], *p* = 0.003).

**TABLE 4:** Adjusted odds ratios identifying significant predictors of self‐reported hair loss in users of weight loss.

Predictor	Adjusted OR	95% CI	*p*‐value
Gender			
Male (vs. Female)	0.36	[0.18, 0.70]	0.003
Marital Status			
Single (vs. Divorced)	6.83	[1.41, 33.15]	0.017
Married (vs. Divorced)	9.94	[2.10, 46.96]	0.004
Injection Type			
Mounjaro (vs. Victoza/Saxenda/Ozempic)	3.02	[1.32, 6.91]	0.009
Duration of Injection Use			
< 3 months (vs. > 12 months)	1.04	[0.36, 2.98]	0.940
3–6 months (vs. > 12 months)	2.38	[0.84, 6.74]	0.101
6–12 months (vs. > 12 months)	3.50	[0.99, 12.40]	0.053

Marital status was also a significant predictor. Compared to divorced individuals, single participants had 6.83 times higher odds of reporting hair loss (95% CI [1.41, 33.15], *p* = 0.017), whereas married participants had even greater odds (adjusted OR = 9.94, 95% CI [2.10, 46.96], *p* = 0.004).

The type of injection used emerged as a strong predictor: participants using Mounjaro had significantly higher odds of reporting hair loss compared to those using Victoza, Saxenda, or Ozempic (adjusted odds ratio = 3.02, 95% confidence interval [1.32, 6.91], *p* = 0.009).

Regarding the duration of injection use, those who had been using injections for less than 3 months (adjusted OR = 1.04, 95% CI [0.36, 2.98], *p* = 0.940) or for 3–6 months (adjusted OR = 2.38, 95% CI [0.84, 6.74], *p* = 0.101) did not show statistically significant associations with hair loss. However, those using injections for 6–12 months showed a trend toward significance, with higher odds of hair loss compared to those using injections for more than 12 months (adjusted OR = 3.50, 95% CI [0.99, 12.40], *p* = 0.053).

## 4 Discussion

Over three‐quarters of participants in our study reported new or worsened hair shedding after initiating GLP‐1RAs. Female participants had substantially higher odds of experiencing hair loss compared to males, a finding consistent with previous studies that suggest women are at a higher risk for drug‐induced alopecia, particularly in the context of physiological stress [8]. This is particularly relevant given that women tend to be more sensitive to hormonal changes, and hair loss in females is often more noticeable and distressing. Furthermore, we found that participants using Mounjaro were about three times more likely to report hair loss compared to those using other GLP‐1RAs. This aligns with pharmacovigilance reports, which suggest that it may be due to its more significant weight‐reducing effects [10, 11]. In our study, 43.4% of Mounjaro users reported severe hair loss, a significantly higher rate compared to Ozempic users, who reported 9.5% severe hair loss. Saxenda users had intermediate rates (42.9%), although the sample size for Saxenda was smaller.

This study suggests that patients using GLP‐1 receptor agonists (GLP‐1RAs) for 3 to 6 months are more likely to experience hair loss, indicating that this period when weight loss is most significant, drug induced alopecia with characteristics similar to telogen effluvium. Participants who had been using GLP‐1RAs for 6 to 12 months showed a trend toward increased hair loss; however, the 3‐ to 6‐month period was identified as particularly susceptible to this side effect. This finding is consistent with the literature, which links rapid weight loss during the first few months of GLP‐1RA therapy to the triggering of hair shedding [2, 12, 13]. Furthermore, the progression of hair loss over time showed that most participants reported hair loss worsening during therapy, with 48.2% indicating an increase in shedding. However, a significant portion (40.3%) reported that their hair loss had stabilized, indicating that in many cases, this drug induced hair loss is similar to telogen effluvium resolves as the body adapts. However, a case report from Saudi Arabia has also documented alopecia areata as a potential adverse effect of GLP‐1 receptor agonists [14]. Additionally, recent studies have highlighted alopecia as a possible dermatologic side effect of GLP‐1RAs, further emphasizing the need to monitor for such adverse events during treatment [15, 16].

Hair loss, even if temporary, can significantly impact a patient's psychological well‐being. Many participants in our study reported moderate to severe shedding, with over one‐third stating that their hair loss worsened during treatment. This mirrors findings from other studies, where alopecia‐related anxiety was identified as a significant concern among patients [8, 17]. Hair loss can lead to emotional distress and decreased self‐esteem, sometimes resulting in early discontinuation of therapy. To mitigate this, healthcare providers should inform patients about the potential for hair loss at the start of GLP‐1RA therapy and reassure them that it is typically reversible. Proper nutritional support, including adequate protein intake and supplementation with iron, zinc, and biotin, is crucial for preventing and managing hair loss. Additionally, screening for underlying conditions, such as thyroid dysfunction, should be considered before starting treatment to support hair health further.

This study has several limitations, including the self‐reported nature of hair loss and the absence of a control group, which makes it challenging to distinguish the effects of weight loss from those of the drug itself. Future research should incorporate objective dermatologic assessments and include a control group of patients using alternative weight‐loss methods better to isolate the specific impact of GLP‐1RAs on hair loss. Additionally, larger studies with more diverse populations are needed to investigate how factors such as gender, age, and genetics affect the risk of hair loss.

## 5 Conclusion

In conclusion, this study highlights the significant association between GLP‐1 receptor agonist (GLP‐1RA) therapy and hair loss, particularly in female patients and those using Mounjaro. Although this drug induced alopecia with characteristics similar to telogen effluvium, is a distressing but typically reversible side effect, it can significantly impact patients' emotional well‐being and adherence to treatment. Proactive management, including patient education, reassurance, and proper nutritional support, is crucial in minimizing the psychological and physical effects of this side effect. Further research, including controlled studies with objective assessments, is necessary to gain a deeper understanding of the underlying mechanisms.

## Author Contributions

Yahya Argobi contributed to the conception, design, and supervision of the study. Norah Saad Jadaan participated in data collection and preliminary analysis. Hind Bader Alshalhoob contributed to the literature review and the design of the methodology. Manar Saleh Alyousef was responsible for interpreting clinical data. Ghaid Mohammed Alotaibi assisted with statistical analysis and data visualization. Hussain Sami Alwesaibie contributed to the validation of the results and the revision of the manuscript. Ibtihal Sultan M. Alshehri participated in manuscript writing and final proofreading. All authors read and approved the final manuscript.

## Ethics Statement

This study was conducted in accordance with the ethical standards of the institutional and national research committees, as well as the 1964 Helsinki Declaration and its subsequent amendments or comparable ethical standards. Ethical approval was obtained from the appropriate ethics review boards of the participating institutions. Informed consent was obtained from all individual participants included in the study.

## Conflicts of Interest

The authors declare no conflicts of interest.

## Data Availability

The data that support the findings of this study are available from the corresponding author upon reasonable request.
